# Polyscore of Non-invasive Cardiac Risk Factors

**DOI:** 10.3389/fphys.2019.00049

**Published:** 2019-02-04

**Authors:** Alexander Steger, Alexander Müller, Petra Barthel, Michael Dommasch, Katharina Maria Huster, Katerina Hnatkova, Daniel Sinnecker, Alexander Hapfelmeier, Marek Malik, Georg Schmidt

**Affiliations:** ^1^Klinik für Innere Medizin I, Technische Universität München, Munich, Germany; ^2^National Heart and Lung Institute, Imperial College London, London, United Kingdom; ^3^Institute of Medical Informatics, Statistics and Epidemiology, Technische Universität München, Munich, Germany

**Keywords:** non-invasive autonomic testing, combination of risk factors, electrocardiogram, blood pressure monitoring, resting respiration, survivors of myocardial infarction, all-cause mortality, low-risk and high-risk group separation

## Abstract

Non-invasive risk stratification of cardiac patients has been the subject of numerous studies. Most of these investigations either researched unique risk predictors or compared the predictive power of different predictors. Fewer studies suggested a combination of a small number of non-invasive indices to increase the accuracy of high-risk group selection. To advance non-invasive risk assessment of cardiac patients, we propose a combination score (termed the Polyscore) of seven different cardiac risk stratifiers that predominantly quantify autonomic cardiovascular control and regulation, namely the slope of heart rate turbulence, deceleration capacity of heart rate, non-invasively assessed baroreflex sensitivity, resting respiration frequency, expiration triggered sinus arrhythmia, post-ectopic potentiation of systolic blood pressure, and frequency of supraventricular and ventricular ectopic beats. These risk stratification tests have previously been researched and their dichotomies defining abnormal results have been derived from previous reports. The Polyscore combination was defined as the number of positive tests among these seven risk predictors, giving a numerical scale which ranges from 0 (all tests normal) to 7 (all tests abnormal). The Polyscore was tested in a population of 941 contemporarily treated survivors of acute myocardial infarction (median age 61 years, 182 females) of whom 72 (7.65%) died during a 5-year follow-up. In these patients, all the risk predictors combined in the Polyscore were assessed during in-hospital 30-min simultaneous non-invasive recordings of high-frequency orthogonal electrocardiogram, continuous blood pressure and respiration. Compared to Polyscore 0 stratum, the hazard ratios of mortality during follow-up increased almost exponentially in strata 1 through 7 (vs. stratus 0, the hazard ratios were 1.37, 1.96, 7.03, 15.0, 35.7, 48.2, and 114, in strata 1 to 7, respectively; *p* < 0.0001). This allowed selecting low-risk (Polyscore ≤ 2), intermediate risk (Polyscore 3 or 4) and high-risk (Polyscore ≥ 5) sub-groups of the population that differed greatly in the Kaplan–Meier probabilities of mortality during follow-up. Since the Polyscore was derived from recordings of only 30-min duration, it can be reasonably applied in different clinical situations including population-wide screening. We can therefore conclude that the Polyscore is a reasonable method for cardiac risk stratification that is ready for prospective validation in future independent studies.

## Introduction

Non-invasive risk stratification of cardiac patients has been addressed in a broad variety of studies (Josephson et al., 1982; Buxton et al., 1984; Wellens et al., 2014). Repeatedly, different risk factors such as left ventricular ejection fraction (LVEF), late potentials, heart rate variability (HRV), and the presence of non-sustained ventricular tachycardia have been compared with the aim of identifying the most powerful risk factor (Farrell et al., 1991). This led, among others, to the present guidelines for the selection of cardiac patients suitable for prophylactic implantation of automatic defibrillators (ICD) (Al-Khatib et al., 2018). These guidelines are based solely on univariable LVEF assessment and are now understood to be far from optimally effective (Køber et al., 2016). It is now also understood that the disappointment with the ICD guidelines cannot be solved by one single test. Rather, it is now believed that a truly effective risk prediction requires a combination of different risk factors.

Powerful cardiac risk predictions beyond LVEF assessment have previously been shown by different tests related to the autonomic nervous system function (Wellens et al., 2014). These tests quantify cardiovascular control at different levels and scales. Some combinations of such tests, e.g., of heart rate turbulence (HRT) and of deceleration capacity (DC) have already been proposed (Bauer et al., 2009) but these proposals have not reflected the wide scope of autonomic regulation. Having this is mind, we are presenting a Polyscore of seven different autonomic and related tests. The risk prediction performance of this Polyscore was evaluated in a well characterized population of survivors of acute myocardial infarction (AMI).

## Materials and Methods

### Population

The population used in the study was previously described (Barthel et al., 2011). Briefly, between May 2000 and March 2005, 941 consecutive AMI survivors were enrolled at two participating high-volume centers (Klinikum rechts der Isar and Deutsches Herzzentrum München, both Technische Universität München, Munich, Germany). The vast majority of the patients (96.8%) were of European Caucasian origin while the remaining few were of mixed descent (Turkish, Arabic, and Asian).

Acute myocardial infarction was diagnosed based on at least 2 of the following findings: typical chest pain lasting ≥20 min, creatine kinase above twice the upper normal limit of the respective laboratory, and admission ST-segment elevation ≥0.1 mV in at least 2 contiguous limb leads or ≥0.2 mV in at least 2 contiguous precordial leads.

Patients were enrolled if aged ≤ 80 years, survived the acute phase of myocardial infarction, were in sinus rhythm, and did not meet the secondary prophylaxis indications for ICD implantation before hospital discharge. The study was approved by the local ethics committee and all participants gave written informed consent.

### Clinical Data

Clinical characteristics were assessed during the hospitalization for the index AMI.

Diabetes mellitus was diagnosed if the patient was already on antidiabetic medication or if fasting blood glucose repeatedly exceeded 11 mmol/l.

Left ventricular ejection fraction was assessed either by echocardiography (biplane Simpson’s rule, Sonos 5500, Hewlett Packard, Palo Alto, CA, United States) or by left ventricular angiography. In compliance with the current ICD-guidelines (Dagres and Hindricks, 2013), LVEF was dichotomized at 35%.

The GRACE (Global Registry of Acute Coronary Events) score (Eagle et al., 2004), a recognized clinical risk score characterizing acute coronary syndrome patients, combines age, serum creatinine, past myocardial infarction, congestive heart failure, in-hospital percutaneous coronary intervention, resting heart rate, systolic blood pressure, ST segment deviation and positive cardiac enzymes. The score ranges from 1 to 210 points and for the purposes of this study was dichotomized at 120 points (Barthel et al., 2012).

### Recordings and Autonomic Tests

During the initial hospitalization and within 2 weeks after the index AMI (median day 7; inter-quartile range 5 to 9 days after the index AMI), simultaneous non-invasive 30-min recordings were performed including an electrocardiogram (1.6 kHz in orthogonal XYZ leads, TMS International, Enschede, Netherlands) and continuous arterial blood pressure by finger photoplethysmographic device (Portapres; TNO-TPD Biomedical Instrumentation, Amsterdam, Netherlands). Respiration was assessed by a piezoelectric thoracic sensor (1.6 kHz, Pro-Tech, Porti system, TMS International). The recordings were made in supine resting position in quiet environment after regular morning medication.

The biosignals were stored digitally and reviewed by experienced technicians blinded to the clinical outcome data. Artifacts were eliminated, and QRS-classifications were manually corrected where necessary.

### Polyscore Components

The following seven previously described risk predictors related to autonomous nervous system function were considered:

(1) HRT, that is the development of RR intervals following a ventricular premature contraction, is one of the well-known risk predictors (Schmidt et al., 1999). Of the two HRT components, turbulence slope appears to be a stronger risk predictor compared to turbulence onset. Therefore, it was used in Polyscore design together with the previously established dichotomy of 2.5 ms per RR interval (Bauer et al., 2008).
(2) DC quantifies vagal effects on the heart by measuring and averaging deceleration-related modulations of the heart rate (Bauer et al., 2006). The optimum dichotomy was previously defined at 2.5 ms average beat-to-beat RR-interval prolongation.
(3) Baroreflex is a cardiovascular control mechanism that prevents excessive blood pressure fluctuations. It increases and decreases heart rate and vascular resistance in response to the decrease and increase in the arterial blood pressure, respectively. The extent of the reflex was quantified by studying the heart rate deceleration following a blood pressure rise in the simultaneous ECG and blood pressure recording by bivariate phase-rectified signal analysis. It was expressed by the baroreflex sensitivity measured in ms of RR interval change per mmHg of blood pressure change. The optimum dichotomy was previously defined at 1.58 ms/mmHg (Barthel et al., 2012).
(4) Average respiration rate was measured during the last 10 min of the recordings when the conditions were fully stabilized. The previously suggested dichotomy limit of ≥18.6 breaths per minute was used to define abnormally fast resting respiration (Barthel et al., 2013).
(5) Expiration-triggered sinus arrhythmia was assessed by studying the RR interval changes during the early expiration phase by bivariate phase-rectified signal analysis. Dichotomy ≤0.19 ms of RR changes was used to define an abnormal heart rate response (Sinnecker et al., 2016).
(6) Systolic blood pressure reaction to single ventricular ectopic beats was expressed by the previously proposed post-ectopic potentiation (PESP). This was quantified by the ratio of the systolic blood pressure of the first post-ectopic beat relative to the systolic pressure values of the following sinus rhythm cycles. Previously published dichotomy of 1.03 was used to define presence or absence of PESP (Sinnecker et al., 2014).
(7) Ectopic beats have long been recognized as a risk factor in its own right (Moss et al., 1979). Since the source recordings of this study were relatively short, the presence of both supraventricular ectopics (>7 per 30 min) or ventricular ectopics (>29 per 30 min) were considered to signify abnormal substrate. The dichotomy limits were derived in a retrospective analysis by means of long-rank statistics optimization with the aim of establishing a strong mortality predictor.

### Polyscore Definition

The seven risk factors described in the previous section were evaluated in each study patient. Using their dichotomy limits, each factor was classified as normal or abnormal. Subsequently, the Polyscore was defined as the number of abnormal factors. This led to 8 possible values ranging between 0 (all risk factors normal) and 7 (all risk factors abnormal).

### Follow-Up and Outcome Events

Study patients were followed up with clinical visits every 6 months. In each patient, follow-up was completed after 5 years. Patients who did not attend a scheduled visit were contacted either by letter, telephone or through their general practitioner. In case a patient could not be traced, the population registry was used to identify those who died.

While the follow-up information allowed to classify the death cases into non-cardiac, cardiac non-sudden, and sudden cardiac deaths, total mortality over the 5-year follow-up period was used as the outcome event for the purposes of this study.

### Statistics and Data Presentation

The distribution of continuous data is presented as medians with inter-quartile ranges, categorical data are presented as absolute and relative frequencies.

To study the contribution of individual Polyscore components to the total Polyscore, the relative number of patients who had a given risk factor positive within the groups with Polyscore φ = 1, 2, …, 7 was calculated and displayed graphically.

Univariable Cox regression model was used to calculate hazard ratios (including its 95% confidence intervals) of patients with Polyscore φ vs. Polyscore 0, ranging the value of φ from 1 to 7. These hazard ratios were displayed graphically with the aim of selecting Polyscore cut-off points defining low-risk, intermediate, and high-risk sub-populations.

Using these Polyscore cut-off points, Kaplan–Meier survival curves were calculated for the low-risk, intermediate, and high-risk subpopulations. Log-rank test was used to compare the Kaplan–Meier survival curves. The analysis was subsequently repeated in patients with and without the diagnosis of diabetes mellitus.

Univariable and multivariable Cox regression models were used to compare the strength of risk prediction by LVEF, presence of diabetes mellitus, GRACE score, and the Polyscore. These Cox regression models were used twice, (**a**) using the non-dichotomized values of LVEF, GRACE score and Polyscore (only the presence of diabetes used as a categorical variable) and (**b**) using dichotomized values of all variables.

Kaplan–Meyer survival curves were calculated together with their 95% pointwise confidence intervals using the R statistical package version 3.3.2 (R Core Team, 2018) with the survival package version 2.38 (Therneau, 2018). Other statistical analyses were made using the SPSS package (IBM SPSS Statistics, version 25, Armonk, NY, United States). Hypothesis testing was performed on two-sided 5% significance levels.

## Results

Clinical characteristics of the study population are shown in Table 1. Of the 941 study patients, 11 (1.2%) were lost during the follow-up period and subsequently censored at the time of last contact.

**Table 1:** Clinical characteristics of the study population.

Number of patients	941
Age (years), median (IQR)	61 (52–69)
Females, n (%)	182 (19.3)
Diabetes mellitus, n (%)	184 (19.6)
Hypertension, n (%)	682 (72.5)
Previous or active smoking, n (%)	488 (51.9)
Family history of CAD or stroke, n (%)	281 (29.9)
History of previous MI, n (%)	90 (9.6)
COPD, n (%)	39 (4.1)
CK max (U/l), median (IQR)	1,302 (647–2,465)
LVEF (%), median (IQR)	53 (45–60)
BMI (kg/m^2^), median (IQR)	27 (24–29)
Serum creatinine (md/dl), median (IQR)	1.1 (0.9–1.3)
eGFR_(MDRD)_ ≤ 60 SI units	201 (21.4)
Cardiogenic shock/CPR, n (%)	41 (4.4)
Intervention	
PCI, n (%)	878 (93.3)
Thrombolysis, n (%)	14 (1.5)
CABG, n (%)	6 (0.6)
Aspirin, n (%)	913 (97.0)
Betablockers, n (%)	897 (95.3)
ACE-inhibitors, n (%)	885 (94.0)
Statins, n (%)	879 (93.4)
Diuretics, n (%)	415 (44.1)

*ACE, angiotensine converting enzyme; BMI, body mass index; CAD, coronary artery disease; CABG, coronary artery bypass graft; COPD, chronic obstructive pulmonary disease; CK, serum creatine kinase; CPR, cardiopulmonary resuscitation; eGFR, estimating glomerular filtration rate; IQR, interquartile range; LVEF, left ventricular ejection fraction; MI, myocardial infarction; PCI, percutaneous coronary intervention*.

**Figure 1:**
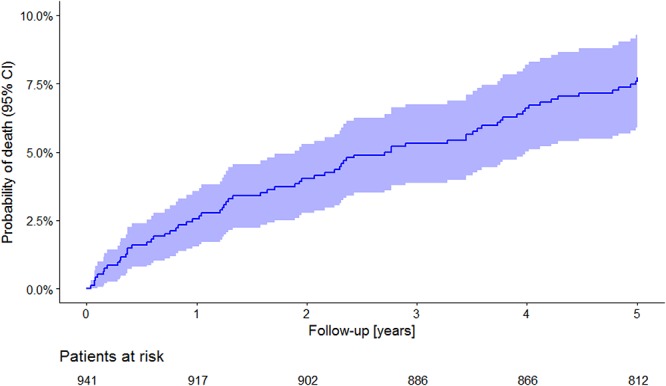
All-cause mortality in the study population. The Kaplan–Meier curve of probability of death is shown together with its 95% confidence intervals. Numbers of patients at risk are shown below the time axis.

During the 5-year follow-up period, 72 (7.65%) patients died. In concordance with the observations made in other post-AMI registries (Farrell et al., 1991) the incidence of death was highest during the first year of follow-up. In subsequent years, the incidence of death was practically constant, and the corresponding Kaplan–Meyer curve was almost linear (Figure 1).

As expected, the numbers of patients in the different Polyscore strata were decreasing from the low-risk to the high-risk strata. The numbers of patients with Polyscore 0 to 7 were 239 (25.4%), 261 (27.7%), 182 (19.3%), 133 (14.1%), 86 (9.1%), 24 (2.6%), 9 (1.0%), and 7 (0.7%), respectively.

The incidence of mortality in the Polyscore strata was gradually increasing from the low-risk stratum 0 to the high-risk stratum 7. The numbers of patients who died during follow-up in the individual Polyscore strata 0 to 7 were 4 (1.7%), 6 (2.3%), 6 (3.3%), 15 (11.3%), 19 (22.1%), 11 (45.8%), 5 (55.6%), and 6 (85.7%).

**Figure 2:**
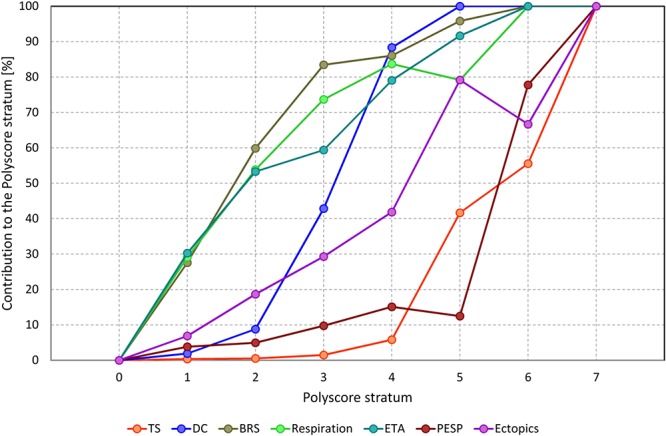
Contribution of individual elements of Polyscore to different Polyscore strata. For each stratum of Polyscore and for each individual risk factor, the graph shows the percentage of patients of the given Polyscore strata in whom this risk factor was positive. Note that the different contribution profiles allow approximate distinction of three groups of risk factors with BRS, respiration frequency, and ETA being more sensitive; TS and PESP being more specific; and DC and ectopic frequency in-between (see the text for further details). TS, turbulence slope; DC, deceleration capacity; BRS, baroreflex sensitivity; Respiration, average respiration frequency; ETA, expiration triggered sinus arrhythmia; PEST, post-ectopic systolic blood pressure potentiation; Ectopics, frequency of ventricular or supraventricular ectopic beats.

Figure 2 shows the contribution of separate risk factors to the individual Polyscore categories. Three groups of risk factors can be distinguished. While the absence of abnormalities in BRS, respiration frequency, and ETA tends to signify overall low risk, the abnormalities in turbulence slope and PESP tend to indicate high risk. These are complemented by DC and ectopic frequency that gradually appear more frequently with increasing Polyscore strata.

**Figure 3:**
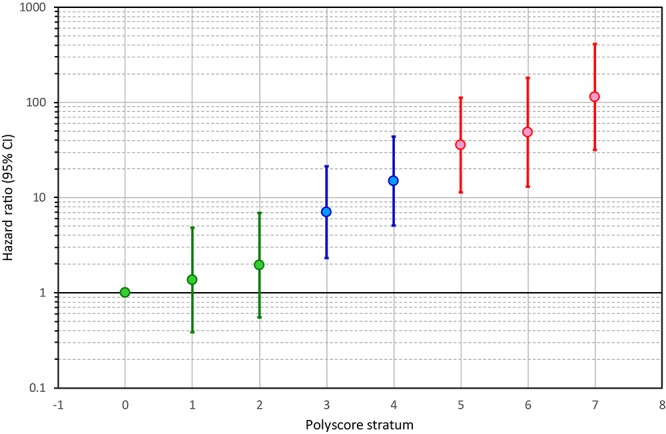
Hazard ratios of individual Polyscore strata. For each stratum 1, 2, …, 7, the figure shows the result of a univariable Cox regression model comparing the stratum with the lowest-risk stratum of Polyscore 0. The Hazard ratios are shown together with their 95% confidence intervals. Note the logarithmic scale of the vertical axis. Note also that the development of the hazard ratios allows defining a low-risk group of Polyscore ≤ 2 (shown in green), an intermediate risk group of Polyscore 3 or 4 (shown in blue), and a high-risk group of Polyscore ≥ 5 (shown in red). CI, confidence interval.

This distinction between low-risk and high-risk sub-strata was reflected in the development of univariable hazard ratios of individual Polyscore categories. Figure 3 shows that from Polyscore stratum 1 to stratum 7, the hazard ratios (in comparison to Polyscore stratum 0) increased almost exponentially, *p* < 0.0001, Wald = 127 (note the logarithmic scale of the vertical axis of the figure). This allowed to select Polyscore dichotomies separated by similar hazard ratio increases. Specifically, hazard ratio of Polyscore 3 (vs. Polyscore 0) was close to 6 (exactly 7.027). Another 6-times hazard increase (to hazard ratio of 6^2^ = 36) was very close to the hazard ratio of Polyscore 5 (exactly 35.71; still vs. Polyscore 0). We have therefore dichotomised Polyscore at ≤2 to define a low-risk group, and at ≥5 to define a high-risk group. Patients with Polyscore categories 3 and 4 constituted an intermediate risk group.

**Figure 4:**
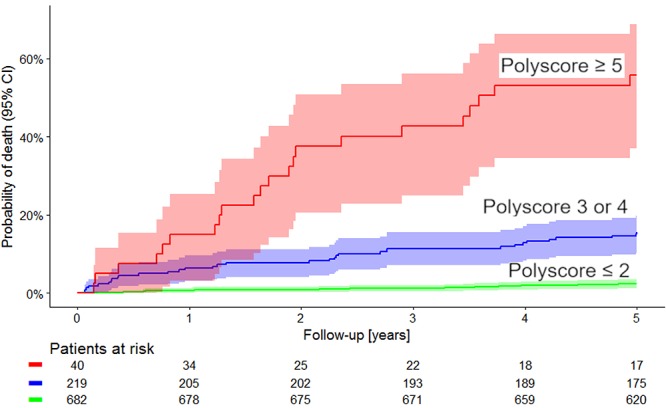
Comparison of Kaplan–Meier probabilities of death in the population sub-groups defined by Polyscore ≤ 2 (green), Polyscore 3 or 4 (blue) and Polyscore ≥ 5 (red). Albeit positively biased (see the text for details) the difference between the probabilities of death in these groups was statistically significant (*p* < 0.0001, χ^2^ = 220). Numbers of patients at risk in the individual sub-groups are shown below the time axis.

Kaplan–Meier survival curves of these risk categories are shown in Figure 4. The figure shows that the probabilities of death were clearly separated between the low, intermediate, and high-risk groups. This is not surprising since our selection of the sub-groups based on the results shown in Figure 3 made the differences between these sub-groups positively biased. Hence, although we also calculated the statistical comparison of the Kaplan–Meier curves (*p* < 0.0001, χ^2^ = 220) the biased nature of this test needs to be pointed out.

**Figure 5:**
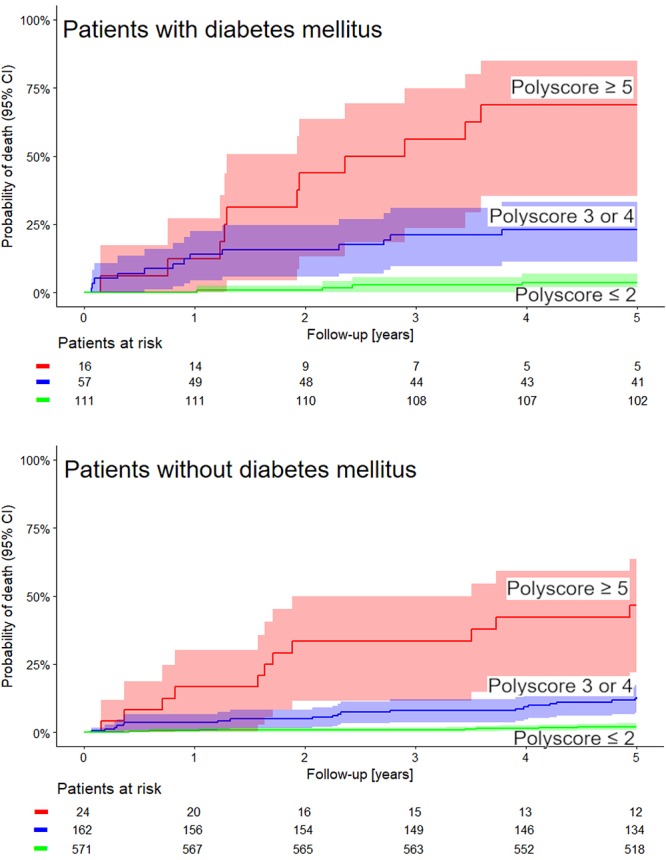
The same comparison of Kaplan–Meier probabilities of death as shown in Figure 4 repeated separately for patients with the diagnosis of diabetes mellitus (top panel) and for patients without the diagnosis of diabetes mellitus (bottom panel). In both cases, the differences between the probabilities of death in these groups was statistically significant (χ^2^ = 59 and χ^2^ = 123, respectively, both *p* < 0.0001). Numbers of patients at risk in the individual sub-groups are shown below the time axes.

The univariable and multivariable Cox models comparing the predictive power of Polyscore in comparison with LVEF, presence of diabetes mellitus, and GRACE score are shown in Tables 2, 3. In both the non-dichotomized and dichotomized versions of the Cox models, the Polyscore led to the largest χ^2^ values and to the largest hazard ratios. In particular, while the Polyscore is predominantly related to the assessment of cardiac autonomic status, the risk prediction by Polyscore was independent of and stronger than that by the presence of diabetes mellitus which is a known source of autonomopathies in cardiac patients (Barthel et al., 2011; May et al., 2018; Yang et al., 2018). This was confirmed by the comparisons of Kaplan–Meier survival curves of dichotomized Polyscrore risk categories in patients with and without diabetes mellitus as shown in Figure 5.

**Table 2:** Cox regression model using non-dichotomised variables.

Variable	Univariable model			Multivariable model		
	HR	χ^2^	*p*	HR	χ^2^	*p*
LVEF (%)	0.96 (0.94 – 0.97)	24.2	<0.0001	0.99 (0.98 – 1.01)	0.5	0.473
Diabetes (yes/no)	2.78 (1.73 – 4.47)	17.9	<0.0001	1.60 (0.99 – 2.60)	3.7	0.473
GRACE score	1.04 (1.03 – 1.05)	65.0	<0.0001	1.02 (1.01 – 1.03)	13.3	<0.0001
Polyscore	2.12 (1.86 – 2.41)	127.1	<0.0001	1.78 (1.53 – 2.07)	55.9	<0.0001

*HR, hazard ratio shown together with 95% confidence interval; LVEF, left ventricular ejection fraction*.

**Table 3:** Cox regression model using dichotomised variables.

Variable	Univariable model			Multivariable model		
	HR	χ^2^	*p*	HR	χ^2^	p
LVEF ≥ 35%	4.29 (2.56 – 7.19)	30.6	<0.0001	1.72 (1.00 – 2.96)	3.8	0.050
Diabetes (yes/no)	2.78 (1.73 – 4.47)	17.9	<0.0001	1.73 (1.06 – 2.80)	4.9	0.027
GRACE score ≥ 120	5.82 (3.41 – 9.92)	41.9	<0.0001	2.62 (1.47 – 4.66)	10.8	0.001
Polyscore ≥ 5	35.71 (11.36 – 112.24)	113.2	<0.0001	15.65 (7.62 – 32.17)	56.0	<0.0001

*HR, hazard ratio shown together with 95% confidence interval; LVEF, left ventricular ejection fraction*.

## Discussion

The study shows that a combination of different non-invasive risk predictors can be combined into a powerful Polyscore that, at least in the data of the present investigation, outperforms other previously established risk stratification techniques. The success of the Polyscore is most likely related to its multifactorial nature. The constituent risk factors characterize different facets at different scales of cardiovascular as well as other regulation and control. Whilst some of these factors are related to the autonomic control of the sinus nodal periodicity, others quantify respiration and its influence on the autonomic (mainly vagal) reflexes, blood pressure regulation, and vascular responsiveness.

The vast majority of previous studies of post-AMI risk stratification were based on HRV and other related autonomic indices and utilized long-term recordings of nominal 24-h duration (Kleiger et al., 1987; Farrell et al., 1991). Such a prolonged period of recording allowed studying not only the day-night differences but also to quantify the autonomic responsiveness of the organism to different environmental stimuli and challenges. For these reasons, it was necessary to reasonably standardize the recording conditions which led to the need of recording the AMI survivors before hospital discharge (Mäkikallio et al., 2005). In truly ambulatory conditions, the differences in the surrounding inputs and activity of different patients resulted in far too substantial inter-subject variability of autonomic measurements (Yoshizaki et al., 2013) which prevented meaningful definitions of low- and high-risk subgroups. Subsequently, it became recognized that the necessity of prolonged monitoring of in-patients is a healthcare burden that negatively impacted on the practical utility of HRV and of some associated autonomic risk indicators (Huikuri et al., 2017). This contrasts with the design of the study that provided the data analyzed here. The Polyscore is based on indices derived from controlled 30-min recordings in undisturbed supine position. Thus, little effects of external challenges are involved and the Polyscore reflects the conditions of intra-organism homeostasis control. The limit of 30 min also makes the Polyscore assessment procedures practical for studies in different populations including those of out-patients.

As shown in Figure 2, there are differences in the performance of the risk factors that constitute the Polyscore. Although all the contributing risk factors (apart from ectopic frequency) quantify autonomic reflexes and control mechanisms, their different scales and control reflexes allow to combine factors that are differently sensitive to the mortality risk. Consequently, the Polyscore allows definition of both low-risk and high-risk subpopulations that differ very substantially in their outcomes.

Combination of different risk factors including autonomic indices is not entirely new and other possible combinations have previously been attempted. Among others, the DINAMIT study selected patients with reduced LVEF, increased heart rate, and reduced 24-h HRV (Hohnloser et al., 2004). Similar to other studies (Arisha et al., 2013), the design of the REFINE-ICD trial (Perkiömäki et al., 2015) included the combination of abnormal HRT and of T wave alternans (TWA). Our teams have previously showed improved risk stratification based on the combination of HRT and DC (Bauer et al., 2009). Nevertheless, in these and other similar approaches, the number of combined autonomic indices was small, and studies aimed at improving high-risk group predictions rather than developing a system classifying the patients into different risk strata. This possibility of defining separate groups with gradually increasing risk is an obvious advantage of the Polyscore concept. Purposefully, we have therefore selected two dichotomy limits of ≤2 and ≥5 points of Polyscore since a selection of a singular limit cannot meaningfully define both very low- and very high-risk groups.

Whilst the source data of the study were collected already during the previous decade, the population clinical characteristics shown in Table 1 demonstrate that the patients received treatment consistent with present standards. Among others, more than 90% of the patients were treated by acute coronary intervention. Similarly, the vast majority received beta-blockers, ACE-inhibitors, aspirin, and statins. The source data thus correspond to the contemporary clinical standards which makes the Polyscore ready for prospective applications.

The strength of the Polyscore in the Cox regression analysis was undoubtedly contributed by the retrospective nature of the study and by using previously optimized dichotomies of the individual constituents. Nevertheless, the gradual increase of the hazard ratios associated with individual Polyscore categories proves the credibility of the concept. Naturally, all retrospective studies are mainly hypothesis generating but the strengths of the Polyscore make it a strong candidate for future prospective testing both in post-AMI patients and in other clinically well-defined clinical populations. The practicality of the Polyscore that we already discussed makes it also suitable for cardiac risk screening in general population especially if a system is set-up allowing computerized evaluation of the individual tests that constitute the Polyscore with no or minimum manual intervention.

### Limitations

Several limitations of our data and of the composite of the Polyscore need to be considered. On purpose, we have restricted the Polyscore mainly to autonomic indices that have been previously investigated. Factors of myocardial repolarization abnormality such as TWA, QT interval variability (Baumert et al., 2016), or spatial QRS-T angle (Hnatkova et al., 2018) might also be combined with other risk indices including the autonomic abnormalities. We can only speculate that the gradual increase of risk in the Polyscore categories would be only marginally improved by adding further factors. The individual risk factors that we combined contributed to the Polyscore equally. It would be possible to optimize their contributions further (e.g., by means of a Cox model) and/or to consider continuous scales of individual Polyscore components. Nevertheless, that would further deepen the retrospective nature of the study. We used all-cause mortality as the primary outcome variable. Other outcomes such as cardiac mortality, reinfarction, stroke, etc., might also be considered in future studies. Since the source population was obtained from a study in AMI survivors, we are unable to comment directly on the strength of the Polyscore in different populations. Nevertheless, because of the general nature of autonomic-related risk predictors, we believe that the concept would be equally applicable to other clinical settings. Intentionally, we restricted the multivariable Cox models to the Polyscore and only three other strong risk factors. Other factors available in the source population and not incorporated in the GRACE score might have also been added (e.g., body mass index, marital/spousal status, post-AMI hypotension). Nevertheless, this might have led to the regression models being overfitted. Finally, since the source population was recorded already during the previous decade, the follow-up period might have been extended, although this might have also weakened the association of the risk factors with the outcome. The mortality probabilities compared over 5 years demonstrate the strength of the Polyscore sufficiently.

## Conclusion and Future Directions

The newly designed Polyscore categorized AMI survivors into strata with almost exponential step-wise increases in the hazard of death during 5-year follow-up. The study also showed that all the individual constituents of the Polyscore contribute meaningfully to the combination. The individual constituent risk factors are also based on well documented physiologic role of autonomic regulation and homeostasis control. The Polyscore can therefore be proposed for prospective validation in future independent data collections.

## Author Contributions

PB, AS, MD, DS, and KMH collected the data experiments. PB, AM, and AS contributed to patient follow-up and study database. GS, PB, and MM conceived and designed the study. AM, PB, MM, GS, KMH, and AH analyzed the data. AS, AM, PB, MD, GS, KH, MM, and AH prepared the manuscript. MM, GS, KH, and KMH controlled and finally approved the manuscript.

## Conflict of Interest Statement

GS owns patents on Heart rate turbulence and on Deceleration capacity. The remaining authors declare that the research was conducted in the absence of any commercial or financial relationships that could be construed as a potential conflict of interest.
